# Transcription-mediated replication hindrance: a major driver of genome instability

**DOI:** 10.1101/gad.324517.119

**Published:** 2019-08-01

**Authors:** Belén Gómez-González, Andrés Aguilera

**Affiliations:** Centro Andaluz de Biología Molecular y Medicina Regenerativa (CABIMER), Universidad de Sevilla-Consejo Superior de Investigaciones Científicas-Universidad Pablo de Olavide., 41092 Seville, Spain

**Keywords:** DNA–RNA hybrids, chromosome fragility, genetic instability, replication fork stalling, transcription

## Abstract

Genome replication involves dealing with obstacles that can result from DNA damage but also from chromatin alterations, topological stress, tightly bound proteins or non-B DNA structures such as R loops. Experimental evidence reveals that an engaged transcription machinery at the DNA can either enhance such obstacles or be an obstacle itself. Thus, transcription can become a potentially hazardous process promoting localized replication fork hindrance and stress, which would ultimately cause genome instability, a hallmark of cancer cells. Understanding the causes behind transcription–replication conflicts as well as how the cell resolves them to sustain genome integrity is the aim of this review.

## Abstract (precis)

In this review, Gómez-González et al. discuss the underlying causes of transcription–replication conflicts, which are major threats to genome integrity. They also discuss mechanisms by which cells resolve these conflicts to sustain genome integrity.

The eukaryotic genome duplicates entirely during each S phase of the cell cycle. For this purpose, each replisome must maintain an accurate rate through the chromatinized DNA template and must overcome frequent obstacles such as DNA lesions resulting from endogenous or exogenous genotoxic sources, proteins tightly bound to DNA, torsional stress, or non-B DNA structures. To achieve such a fundamental task on a faithful and timely manner, eukaryotic cells use interconnected mechanisms to couple DNA replication to DNA damage sensing and repair, hence, counteracting replicative stress and genetic instability. These are hallmarks of tumorigenesis (Hills and Diffley 2014; Gaillard and Aguilera 2016), which gain additional relevance given that cancer risk increases with cell divisions (Tomasetti and Vogelstein 2015), highlighting the role of replication in genetic instability.

At the same time, gene expression is necessary for cell survival and proliferation, transcription potentially being the major source of obstacles faced by an advancing replisome. Despite the temporal or spatial separation between replication and transcription of a number of genes, both processes will inevitably occur on the same DNA region at the same time in certain occasions, causing transcription–replication (T–R) conflicts, as has been extensively reviewed recently in both prokaryotes and eukaryotes (Merrikh et al. 2012; García-Muse and Aguilera 2016; Hamperl et al. 2017). Indeed, mounting evidence supports the proposal that transcription is a major source of genetic instability (Aguilera 2002; Gaillard et al. 2013), an important part of such transcription-associated instability being dependent on DNA replication in eukaryotes (Prado and Aguilera 2005; Gottipati et al. 2008; Paul et al. 2013; Hamperl and Cimprich 2016). Furthermore, replication forks emanating from new replication origins induced by oncogene activation cause T–R conflicts (Jones et al. 2013; Macheret and Halazonetis 2018). These forks are prone to collapse, leading to double-strand breaks (DSBs), suggesting that transcription might be an important source of replicative stress associated with oncogene activation (Jones et al. 2013; Macheret and Halazonetis 2018). Along this line, oncogenesis has been related to the genetic instability created by the increased transcriptional activity at genes induced by oncogenes, such as estrogen-induced genes in breast cancer cells with estrogen overproduction (Stork et al. 2016) or oncogenic RAS overexpression (Kotsantis et al. 2016).

T–R encounters that compromise genome integrity do not necessarily have to rely on a physical collision between both machineries. Since both transcription and replication processes deeply affect topology, chromatin organization and the structure of the DNA template, different mechanisms exist by which transcription compromises genome integrity in a replication-mediated manner. Understanding the causes behind T–R conflicts as well as how the cell resolves them to sustain genome integrity is the aim of this review, focusing mainly on eukaryotes.

## Obstacles to replication fork progression

**Figure 1.**
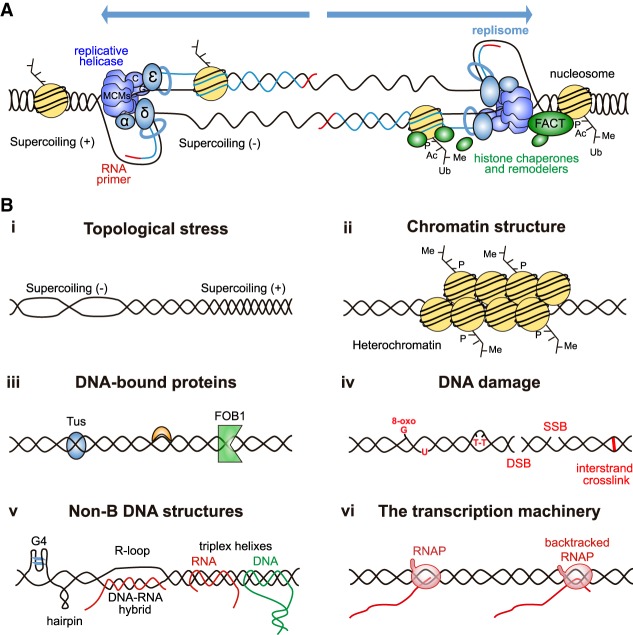
Replication fork progression and obstacles. (*A*) A simplified version of replication forks moving away from a replication origin. Replisomes contain the CMG (MCMs, Cdc45, and GINS) replicative helicase, polymerases α, δ, and ε, and a plethora of additional factors that ensure fork progression, such as histone chaperones (as exemplified by FACT) and remodelers. (*B*) Obstacles to replication fork progression. Fork progression can be hampered by topological stress (panel *i*); certain chromatin structures such as heterochromatin (panel *ii*); other nonnucleosomal DNA-bound proteins (panel *iii*), as exemplified by the Tus protein in bacteria or FOB1-mediated fork barriers in the yeast rDNA; DNA damage, ranging from single-strand breaks (SSBs) and DSBs to interstrand cross-links (ICLs) or base modifications (panel *iv*); non-B DNA structures, including G quadruplexes (G4), hairpins, DNA–RNA hybrids, and R loops as well triplex or cruciform nucleic acid structures that can contain DNA and RNA (panel *v*); and the transcription machinery itself (panel *vi*).

Replication initiates bidirectionally from a well-defined and usually single origin in bacteria but from multiple and less-well-defined origins in eukaryotes. DNA unwinding by the 3′–5′ replicative helicase is coupled to DNA synthesis initiated by the Pol α-primase complex and extended by the leading and lagging strand polymerases (Pol ε and Pol δ, respectively) (Fig. 1A). These proteins work together with a plethora of additional factors that assemble in the structure of the replisome until replication is terminated at chromosome ends or when two forks converge (Bell and Labib 2016). Replication forks, however, can encounter different obstacles during their progression, that can compromise genome integrity if not properly resolved (Fig. 1B).

During fork progression, unwinding of the parental DNA generates the accumulation of topological stress in the form of positive supercoiling (overwinding) ahead. Positive supercoiling would obstruct further unwinding and fork progression, and therefore needs to be counteracted either passively or actively (Fig. 1B, panel i). Passively, fork rotation can alleviate the positive supercoiling but translates it into intertwines between the two new sister chromatids behind the fork, known as DNA precatenates (Postow et al. 1999, 2001) that need to be repaired to enable sister chromatid separation in mitosis (Lucas et al. 2001). Furthermore, excessive fork rotation can induce genomic instability (Schalbetter et al. 2015). Therefore, there is the need for specialized enzymes, DNA topoisomerases, to actively relieve the topological tensions. Topoisomerase function relies on the passing of one DNA molecule through the other by transient single-stranded (type I) or double-stranded (type II) DNA breaks (for review, see Keszthelyi et al. 2016; Pommier et al. 2016). Whereas both Topoisomerase 1 and 2 can act ahead of the fork, the resolution of precatenates accumulated behind the fork requires the specific action of Top2 (Lucas et al. 2001; Cebrián et al. 2015). Importantly, however, accessibility of DNA topoisomerases ahead of the fork is restricted to certain genomic contexts, including when two forks converge (replication termination regions), heterochromatin and other topological barriers such as the nuclear envelope. In these cases, further fork progression is thought to rely exclusively on fork rotation to solve the topological stress (Keszthelyi et al. 2016).

Moreover, DNA replication takes place in the context of a chromatinized DNA template, chromatin being potentially an obstacle to progression. Eukaryotic DNA is wrapped around histone octamers (each containing two copies of each of the four core histones: H2A, H2B, H3, and H4) that are further stabilized by the linker histone H1 into higher-order structures. Histones are marked by posttranslational modifications, such as histone acetylation, methylation, phosphorylation, or ubiquitination, which define the state of the chromatin (for review, see Alabert and Groth 2012). Chromatin has an impact on both replication initiation and fork progression (Alabert and Groth 2012) by either facilitating or making it more difficult to replicate, this being in some cases associated with heterochromatin (Fig. 1B, panel ii; Janssen et al. 2018). Indeed, recent in vitro experiments with reconstituted chromatin have confirmed that fork progression requires accessory factors such as chromatin remodelers and histone chaperones (Devbhandari et al. 2017; Kurat et al. 2017).

Advancing forks can encounter other intrinsically difficult to replicate regions, at which DNA unwinding can be aided by additional helicases, such as the PIF1 family of helicases, which can aid in fork progression through nonnucleosomal DNA-bound proteins (Ivessa et al. 2003). DNA-bound proteins may constitute a transient or full block to DNA unwinding and replisome progression (Fig. 1B, panel iii). Indeed, they can act as barriers ensuring replication termination at specific sites, such as the Tus protein of *Escherichia coli* (Hill et al. 1989; Willis et al. 2014) or Fob1 at the *Saccharomyces cerevisiae* rDNA repeats (Kobayashi 2003). In addition, and despite the existence of multiple DNA repair mechanisms to counteract DNA damage throughout the cell cycle, advancing replisomes can still encounter a damaged template, ranging from single-strand breaks (SSBs) and DSBs to interstrand cross-links (ICLs) or base modifications unable to be copied by the replicative DNA polymerases (Fig. 1B, panel iv). Moreover, replication forks can directly or indirectly stall at non-B DNA structures, including DNA–RNA hybrids, R loops, or G quadruplexes (G4) (Fig. 1B, panel v). The presence of DNA repeats in a sequence can also make it prone to form hairpins, as well as triplex or cruciform nucleic acid structures that impair fork progression (Fig. 1B, panel v; for review, see Mirkin 2006).

In most cases, replication fork blockage in vivo does not occur alone. Thus, the displaced strand in an R loop facilitates the formation of DNA hairpins (Loomis et al. 2014) or G4 structures (Duquette et al. 2004), as open chromatin makes the DNA template more susceptible to DNA damaging agents (Falk et al. 2008) and highly negatively supercoiled DNA enhances the action of damaging agents (LaMarr et al. 1998) and the accumulation of secondary structures (Baaklini et al. 2008). Similarly, the negative supercoiling frequently associated with GC-rich and skewed sequences would potentially promote the formation of G4 structures and DNA–RNA hybrids (Ginno et al. 2013; Manzo et al. 2018).

In addition to these features, accumulating evidence supports that transcription is likely the major source of replicative impairments (Fig. 1B, panel vi). Original studies reported that a fork pauses when encountering transcription in bacterial systems in vitro (Liu and Alberts 1995) and in vivo (French 1992; Mirkin and Mirkin 2005) as well as in yeast (Prado and Aguilera 2005). Furthermore, only S-phase transcription caused genome instability measured as hyper-recombination in yeast (Prado and Aguilera 2005). Consistently, genome-wide analysis revealed that replication forks frequently pause at transcribed units (Azvolinsky et al. 2009). Since transcription affects chromatin, DNA supercoiling and structure, genotoxic accessibility, and non-B DNA structure formation, understanding how transcription impairs fork progression requires tackling the way transcription-associated events contribute to fork stalling.

## The transcription machinery and its potential to stall replication

**Figure 2.**
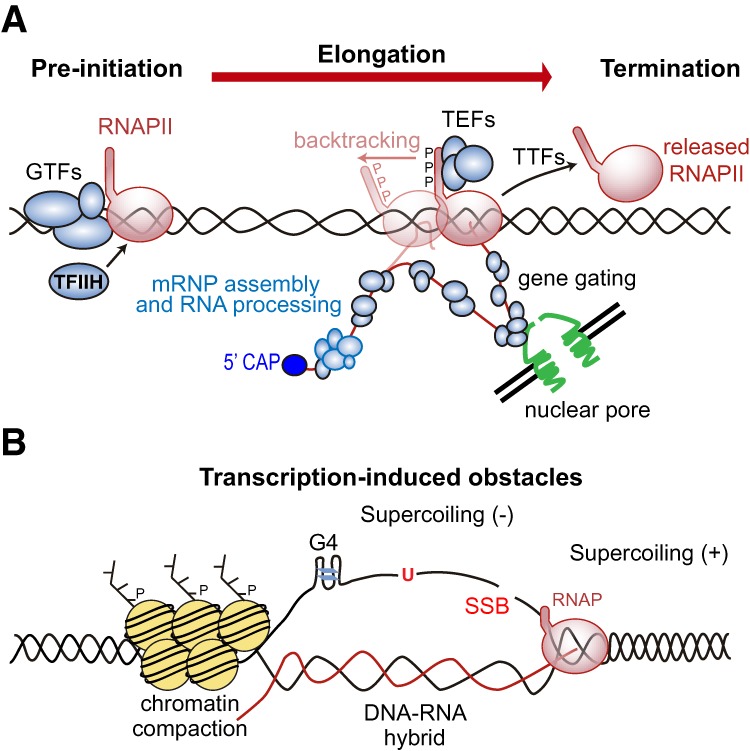
Transcription and its potential to stall replication. (*A*) The RNAPII transcription cycle. RNAPII at its pre-initiation stage sits on DNA with GTFs waiting to be activated by TFIIH. Once activated, elongating RNAPII is ready to synthetize the RNA with the help of TEFs. The RNA is then cotranscriptionally processed into an export-competent mRNP, with gene gating facilitating transcription–export coupling. During elongation, RNAPII pauses at regulatory regions and can even backtrack. Once terminated, RNAPII is released from the DNA. (*B*) Transcription-induced obstacles. In addition to the transcription machinery itself, which is bound to DNA and could block fork progression, transcription enhances the occurrence of structures that hamper replication fork progression. Transcription elongation causes accumulation of positive supercoiling ahead of and negative supercoiling behind the RNAP, enhances the probability of DNA damage, or can promote the formation of non-B DNA structures such as G4 or DNA–RNA hybrids, which have been associated with chromatin compaction.

Actively transcribing genomes are covered by large machineries consisting of different RNA polymerases (RNAPs), transcription- and chromatin-modifying factors, and the nascent RNA. Initiation of transcription is preceded by the loading of a number of general transcription factors (GTFs) before the RNAP is recruited. RNAPII, responsible for protein-coding genes and most noncoding RNAs, is recruited to the promoter as part of the transcription preinitiation complex in its closed form, waiting to be activated by the TFIIH-mediated melting of the DNA and phosphorylation of the C-terminal domain (CTD) of the largest subunit of RNAPII holoenzyme to initiate RNA synthesis. After synthesizing a short transcript, the RNAPII undergoes a promoter-proximal pausing to enable RNA 5′ end capping. Phosphorylation of Ser2 restores transcription and allows the loading of transcription elongation factors (TEFs) and RNA processing factors for productive elongation (Fig. 2A; for review, see Bentley 2014). Transcription elongation is coupled to mRNA packaging and splicing until it reaches the termination region, in which RNA 3′ end processing and termination factors are loaded to generate an export-competent messenger ribonucleoprotein particle (mRNP) and remove the RNAPII from the DNA template. There are several different and often-redundant pathways to ensure proper termination, mostly involving RNAP pausing, RNA cleavage, and/or destabilization of the RNAPII–DNA interaction (for reviews, see Porrua et al. 2016; Proudfoot 2016). In addition to the canonical transcription termination processes, RNAPII transcription can conclude by a roadblock caused by DNA-bound proteins, a mechanism that resembles RNAPI termination and that seems to occur more often than previously foreseen (Candelli et al. 2018). Importantly, the occurrence of transcription at the proximity of the nuclear pores facilitates RNA export through coupling both processes (Fig. 2A; Luna et al. 2008). The complexity of the transcription machinery, together with multiple mRNA processing steps, including 5′ and 3′ end processing and splicing, and mRNP assembly factors may provide a challenge to advancing replication forks. Consequently, cells must have developed specific mechanisms to either avoid or easily resolve T–R conflicts imposed by the threats created at the different transcription stages.

### Fork progression through transcription preinitiation complexes

In principle, an RNAP sitting at the promoter might present a barrier to fork progression much like a protein tightly bound to DNA, but there are not sufficient studies that have tried to specifically evaluate this phenomenon. Genome-wide analysis of replisome positioning in yeast cells have not provided evidence that forks have a major preference to stall at promoter regions (Azvolinsky et al. 2009; Gómez-González et al. 2011; Seo et al. 2012). It is thus possible that unless there is an additional structure or element that holds the RNAP tight to the promoter, replication forks efficiently bypass transcription preinitiation complexes or RNAP sitting at promoters. This may suggest that cells have efficient mechanisms to either remove or bypass the RNAP and/or associated GTFs at the preinitiation stage. Indeed, in vitro seminal studies with the T4 replication machinery revealed that an *E. coli* promoter-bound RNAP can be displaced by the fork with the help of a helicase (Bedinger et al. 1983). Although R loops may accumulate at a number of promoter-proximal regions (Ginno et al. 2012), these do not seem to be a major source of replicative stress in that case. However, promoter regulatory regions could have replication-independent tumorigenic potential, as shown in cells with BRCA1 or BRCA2 cancer-associated mutations, which enhanced RNAPII pausing and DNA–RNA hybrids at promoter-proximal regions (Zhang et al. 2017; Shivji et al. 2018).

### Elongating RNAP stalling and backtracking as sources of T–R conflicts

An important difference emerges once the RNAP enters the elongation and termination phases. Next, the nascent RNA strand in the active pocket of the RNAP hybridizes with the template DNA (over a region of at least 9 nucleotides in the case of RNAPII) (Westover et al. 2004), tightly associating the RNAP with the DNA. It is important to note that the RNAP holoenzyme embraces dsDNA (Barnes et al. 2015), and could a priori constitute a block for replisomes approaching from both directions (head-on and codirectional encounters). In principle, an elongating RNAP could be evicted from chromatin as it happens with promoter-bound RNAP. In fact, the *E. coli* replisome can displace head-on-encountered RNAPs from the DNA to allow replisome progression in vitro (Pomerantz and O'Donnell 2010). The relevance of RNAP removal to prevent T–R conflicts is supported by the observation that yeast RNAPII mutants that retain RNAPII at chromatin cause replication problems (Felipe-Abrio et al. 2015). RNAPII removal occurs as a last-resort response to transcription-blocking DNA lesions and at sites of convergent transcription and it involves ubiquitin-mediated degradation of the largest subunit of RNAPII, Rpb1 (Hobson et al. 2012; Wilson et al. 2013a,b). It is therefore possible that removing elongating RNAPs after fork stalling requires an active process in vivo. So far, RNAPII removal after replication stress was genetically shown to implicate the replication checkpoint, the chromatin-remodeling complex INO80C, and the PAF transcription complex in budding yeast (Poli et al. 2016). In *Schizosaccharomyces pombe*, the RNA interference machinery also promotes RNAP release for heterochromatic silencing (Zaratiegui et al. 2011; Castel et al. 2014).

The transcription cycle involves frequent regulatory pauses, mainly at the 5′ and 3′ ends. The cotranscriptional splicing of the nascent RNA as well as changes in supercoiling, chromatin, and other structural elements in the DNA influence transcription elongation. Furthermore, RNAPs can also pause or arrest at damaged DNA sites, thus facilitating the process of transcription-coupled repair (TCR) required for transcription resumption (Gaillard and Aguilera 2013). In general, RNAP pauses are transient unless the RNAP backtracks leading to a more stable structure that involves losing the contact between the RNAP active site and the 3′ end of the nascent RNA molecule (Fig. 2A; Cheung and Cramer 2011). Backtracking is required for proofreading and occurs at specific regulatory regions but can also occur during elongation when encountering a damaged template or chromatin and topological obstacles (for review, see Gómez-Herreros et al. 2012; Nudler 2012). After backtracking, transcription resumption relies on the cleavage of the displaced transcript to restore contact of the RNA 3′ end with the RNAP active site, which occurs with the help of specific factors such as bacterial GreA and GreB (Opalka et al. 2003; Tehranchi et al. 2010) or eukaryotic TFIIS (Cheung and Cramer 2011). Importantly, a backtracked RNAP may constitute a threat to fork progression, leading to genetic instability as shown in bacteria mutated in GreA and GreB (Dutta et al. 2011).

Given the potential harmfulness of backtracking, cells have developed mechanisms to limit it by favoring RNAP elongation, eviction or degradation, which may help to avoid deleterious T–R conflicts (Fig. 2A). Thus, the coupling of transcription and translation in bacteria limits backtracking (Proshkin et al. 2010). On the other hand, specific factors, such as bacterial DskA prevent backtracking by avoiding nucleotide misincorporation (Tehranchi et al. 2010; Roghanian et al. 2015). In human cells, RECQL5, a DNA helicase that interacts with RNAPI and RNAPII, has also been shown to prevent backtracking by promoting transcription elongation. This function of RECQL5 counteracts T–R conflicts directly (Saponaro et al. 2014; Urban et al. 2016), supporting the idea that a backtracked RNAP enhances the probabilities of T–R conflicts in eukaryotic cells.

### Transcription termination

A role for transcription termination factors (TTFs) in preventing T–R conflicts was first reported based on the observation that mutants in the bacterial termination factor Rho led to replication-dependent DSBs (Washburn and Gottesman 2011). Transcription-associated genetic instability and replication defects were later reported in certain yeast termination mutants, such as those with mutations in the RNA 5′ end processing factors Rna14, Rna15, Fip1, or Hrp1 (Luna et al. 2005; Stirling et al. 2012; Gaillard and Aguilera 2014) or in the Xrn2 exoribonuclease (Morales et al. 2016). These studies could suggest that inefficient transcription termination leads to T–R conflicts. Moreover, recent mapping of Okazaki fragments (OK-seq) indicates that paused RNAPII at transcription termination sites serves to drive replication termination (Chen et al. 2019), indicating that T–R conflicts occur at termination regions and could even have a physiological role by contributing to coordinate the orientation of transcription and replication in a codirectional manner.

Likewise, the DNA–RNA helicase senataxin, which as part of the Nrd1–Nab3–Sen1 (NRD) complex is involved in noncoding RNA termination (Proudfoot 2016; Porrua et al. 2016), has a striking role in preventing transcription-associated genetic instability in yeast (Mischo et al. 2011) and human cells (Skourti-Stathaki et al. 2011). However, transcription-associated genetic instability phenotypes were not observed in other NRD mutants (Mischo et al. 2011). Furthermore, the loss of senataxin leads to not only inefficient termination but also the formation of DNA–RNA hybrids that affect fork progression (Mischo et al. 2011; Skourti-Stathaki et al. 2011; Alzu et al. 2012). Several other reports suggest that senataxin has a role beyond canonical transcription termination. Budding yeast senataxin, but not Nrd1, associates with replication forks (Alzu et al. 2012) and is regulated during the cell cycle peaking in S/G2 (Mischo et al. 2018), and its depletion leads to DNA breaks along the chromosomes, as mapped by Rad52 immunoprecipitation (Costantino and Koshland 2018). In addition, immunofluorescence experiments have shown that human senataxin forms foci that associate with DNA damage markers after replication stress (Yuce and West 2013). Altogether, these reports suggest that senataxin could be recruited to solve T–R conflicts, likely through its role as a DNA–RNA helicase but it is also possible that senataxin promotes RNAPII release at T–R conflict sites.

## Transcription as an enhancer of replication obstacles

Transcription can not only obstruct replication fork progression by itself but also enhance the occurrence of structures that impede fork progression by modifying the template DNA structure and topology as well as in the surrounding chromatin (Fig. 2B).

### Transcription-induced DNA damage

Even though transcription can be used to favor repair of RNAP-blocking DNA lesions via TCR (Gaillard and Aguilera 2013), it can also be an important source of DNA damage, leading to transcription-associated genetic instability. This has been demonstrated in bacteria, yeast, and human cells by the induction of mutagenesis and recombination at a particular DNA sequence when it was heavily transcribed, a phenomenon that has been broadly reviewed (Aguilera 2002; Gaillard et al. 2013; Jinks-Robertson and Bhagwat 2014). Although an important part of transcription-associated genetic instability is likely caused by fork stalling caused by the RNAP itself, transcription can also enhance damage directly, this probably being the major cause of transcription-associated mutagenesis. This could be explained by an increased accessibility of the DNA when it is transcribed due to more open chromatin or even to its topological state. Indeed, accumulation of negative supercoiling behind RNAP could lead to transient regions of ssDNA, which is chemically less stable than dsDNA (Lindahl 1993), as well as to damaging agents or DNA-modifying enzymes. Thus, transcription-induced DNA damage can certainly contribute to fork stalling.

### Chromatin alterations

The number of reports on the effects of chromatin context on transcription has extensively grown since it was first discovered that in vitro transcription is impeded by nucleosomes (Knezetic and Luse 1986; Lorch et al. 1987) and that histone modifications affect gene expression in vivo (Han and Grunstein 1988; Kayne et al. 1988). Transcription elongation through chromatin is aided by the histone chaperone FACT as well as by chromatin remodelers, such as RSC, and histone acetyltransferases, such as NuA4 or SAGA (Li et al. 2007). The intricate relationship between transcription and chromatin state is clearly manifested in the distribution along the gene bodies of most histone modifications, which responds to a histone code that cells would interpret differently to exert specific functions (for review, see Li et al. 2007). Thus, transcribed regions (euchromatin) are typically associated with acetylation of histones H3 and H4 as well as to dimethylation or trimethylation of the Lys4 of histone H3 (H3K4me2 or H3K4me3) (Fig. 2A), whereas H3K9me and H3K27me, are often associated with heterochromatic regions. Inevitably, these chromatin marks would influence the ability of a replication fork to pass through.

Several reports have highlighted transcription-associated impacts on chromatin that could potentially hamper fork progression, such as cotranscriptional DNA–RNA hybrids, which induce chromatin compaction (Fig. 2B; Castellano-Pozo et al. 2013; Colak et al. 2014; Groh et al. 2014; Loomis et al. 2014; Skourti-Stathaki et al. 2014) and RNAP pausing, which drives heterochromatin formation (Parsa et al. 2018). On the other hand, yeast and human cells lacking the FACT complex showed transcription-associated genetic instability and fork progression impairments, implying a key role of this histone chaperone to prevent T–R conflicts (Herrera-Moyano et al. 2014). In agreement, the histone chaperones FACT and CAF1 are specifically recruited to transcribing chromatin to promote fork progression (Li et al. 2018). Other chromatin factors could also inherently protect the cell from T–R conflicts by regulating the coordination between DNA replication and transcription, and, indeed, depletion of histone H1, a key heterochromatin component, causes replication stress and DNA damage linked to T–R conflicts in *Drosophila* and human cells (Bayona-Feliu et al. 2017; Almeida et al. 2018).

### Topological constraints

Transcription elongation causes accumulation of positive supercoiling in front and negative supercoiling behind the RNAP, according to the twin supercoiled domain model (Fig. 2B; Liu and Wang 1987). Whereas, as mentioned previously, positive supercoiling obstructs further unwinding, negative supercoiling can destabilize the physiological structure of DNA favoring not only a major susceptibility to DNA damage but also the formation or stabilization of non-B DNA structures. In principle, T–R conflicts would topologically resemble transcription–transcription (convergent genes) or even replication–replication (regions of replication termination) encounters. Indeed, highly transcribed regions seem prone to topological stress (Bermúdez et al. 2010; Kouzine et al. 2013; Naughton et al. 2013). However, convergent transcription does not cause an enhanced detectable increase in genetic instability even in topoisomerase-deficient mutants (García-Rubio and Aguilera 2012; Pannunzio and Lieber 2016) and does not seem to cause a major and detectable topological stress at least at some convergent genes (Naughton et al. 2013). This may mean that the topological constraint by itself is not sufficient to compromise genome integrity and that, certainly, topoisomerases efficiently remove transcription-associated changes in DNA supercoiling, as the bacteria and yeast genetic data suggest (Sternglanz et al. 1981; Drolet 2006; Tuduri et al. 2009; García-Rubio and Aguilera 2012; Joshi et al. 2012).

It would be important to establish up to which point positively supercoiled DNA ahead of an elongating RNAP would constitute a difficult to replicate region without the need of a physical collision between the transcription and replication machineries. Under this scenario, a T–R conflict could putatively induce fork rotation as a way to locally release the torsional stress. However, any failure to properly do so would promote that the accumulated positive supercoiling arrests the fork, potentially leading to fork reversal (see below).

### Cotranscriptional R loops

Non-B DNA structures, such as hairpins, G4 structures, and R loops, consistent in a DNA–RNA hybrid and the displaced ssDNA (Fig. 2B), may block fork progression. Physiological R loops can form regularly at specific regions, such as the S regions of the Immunoglobulin genes (Yu et al. 2003). However, unscheduled R loops are an important source of genetic instability. Cells thus have developed several mechanisms to prevent their accumulation. DNA–RNA hybridization is prevented by RNA processing and export factors, such as the THO complex (Huertas and Aguilera 2003), the ASF/SF2 RNA processing factor (Li and Manley 2005), and others, as reviewed extensively (Aguilera and García-Muse 2012; Santos-Pereira and Aguilera 2015; Aguilera and Gómez-González 2017). These factors would coat the nascent RNA, limiting its capacity to hybridize back with the DNA template. R-loop formation is also limited by the cotranscriptional control of local supercoiling and chromatin structure that would directly impact the availability of the DNA template to hybridize with the RNA (Tuduri et al. 2009; French et al. 2011; Bayona-Feliu et al. 2017; Salas-Armenteros et al. 2017; Taneja et al. 2017; Almeida et al. 2018). Dysfunction of any of these mechanisms would enhance the accumulation of unscheduled R loops.

Several studies have led to the conclusion that the genetic instability associated with cotranscriptional R loops is due to hindrances in fork progression. Replication impairment and genetic instability was detected in most of the R-loop-accumulating cells (Huertas and Aguilera 2003; Li and Manley 2005; Wellinger et al. 2006; Tuduri et al. 2009; Gan et al. 2011; Bayona-Feliu et al. 2017; Salas-Armenteros et al. 2017; Almeida et al. 2018). Replication-induced DNA breaks caused by estrogen-mediated changes in transcription are also R-loop-dependent (Stork et al. 2016). Furthermore, the increased genetic instability associated with head-on T–R conflicts in bacteria and yeast is at least partially dependent on the presence of DNA–RNA hybrids (Lang et al. 2017; García-Rubio et al. 2018) and persistent DNA–RNA hybrids cause DNA breaks preferentially when they occur close to a head-on replication fork (Costantino and Koshland 2018). Strong evidence that R loops block fork progression, thus promoting T–R conflicts and transcription-mediated DNA damage, comes from the fact that replication-associated repair factors, such as FACT, BRCA1, BRCA2, and other members of the Fanconi anemia (FA) pathway are required for repair and proper fork progression through T–R conflict sites and R loops (see below; Bhatia et al. 2014; García-Rubio et al. 2015; Hatchi et al. 2015; Schwab et al. 2015; Madireddy et al. 2016; for review, see Bhatia et al. 2017). The FA pathway has a key role in the repair of ICLs. Although ICLs promote checkpoint signaling independently of replication, their removal during the S/G2 phases of the cell cycle is coupled to DNA replication and depends on the FA pathway (for review, see Constantinou 2012), thus suggesting a role for FA factors in replication-dependent R-loop removal.

Nevertheless, there are several reasons to believe that DNA–RNA hybrids by themselves do not block fork progression. Apart from the harmless DNA–RNA hybrids of Okazaki fragments or of mitochondrial replication initiation regions, replicative helicases can unwind DNA–RNA hybrids as well as duplex RNA in vitro (Shin and Kelman 2006). Furthermore, recent results provide evidence that replication forks can clear codirectionally formed DNA–RNA hybrids in vivo (Hamperl et al. 2017; García-Rubio et al. 2018). Whether this is performed by the replicative helicase itself or aided by accessory helicases remains an open question. A recent study reported specific R-loop-accumulating yeast histone mutants impaired in H3 serine 10 phosphorylation that had no detectable consequences on genome integrity, thus definitely concluding that a second step, likely involving chromatin alterations, is required for DNA–RNA hybrids to be harmful (Fig. 2B; García-Pichardo et al. 2017).

This second step, however, can also be achieved by the binding of DNA–RNA hybrid stabilizing factors independent of chromatin modifications. This is the case for overexpression of the yeast DNA–RNA-binding protein Yra1, which is able to bind ssDNA as well as DNA–RNA hybrids (García-Rubio et al. 2018) or expression of HB-GFP, a hybrid-binding domain of RNaseH fused to GFP, which induced DNA damage in R-loop-accumulating human cells depleted of BRCA2 (Bhatia et al. 2014). Although not formally demonstrated, such DNA damage could be due to fork impairment. The possible existence of proteins that, like Yra1, are capable of stabilizing R loops could be a physiological strategy to control R-loop-driven effects. Indeed, gene expression has been shown to be modulated in *Arabidopsis* by the binding of AtNDX, a homeodomain-containing protein, to the displaced ssDNA of R loops at the *COOLAIR* long noncoding RNA locus (Sun et al. 2013). Although replication through these regions has not been studied, it is conceivable that this or some other R-loop-stabilizing proteins could potentially modulate T–R conflicts. The recent identification of new DNA–RNA hybrid-binding proteins (Cristini et al. 2018; Wang et al. 2018) could possibly shed light on this.

Finally, it is worth noticing that DNA–RNA hybrids are also enhanced by either single- or double-stranded DNA breaks (Roy et al. 2010; Britton et al. 2014; Li et al. 2016; Ohle et al. 2016; Cohen et al. 2018), which suggests that cotranscriptional DNA breaks would also contribute to the formation of unscheduled R loops capable of compromising genome instability (Aguilera and Gómez-González 2017). However, the relevance of such break-induced R loops on DNA replication has not been evaluated.

### Head-on vs. codirectional T–R conflicts

**Figure 3.**
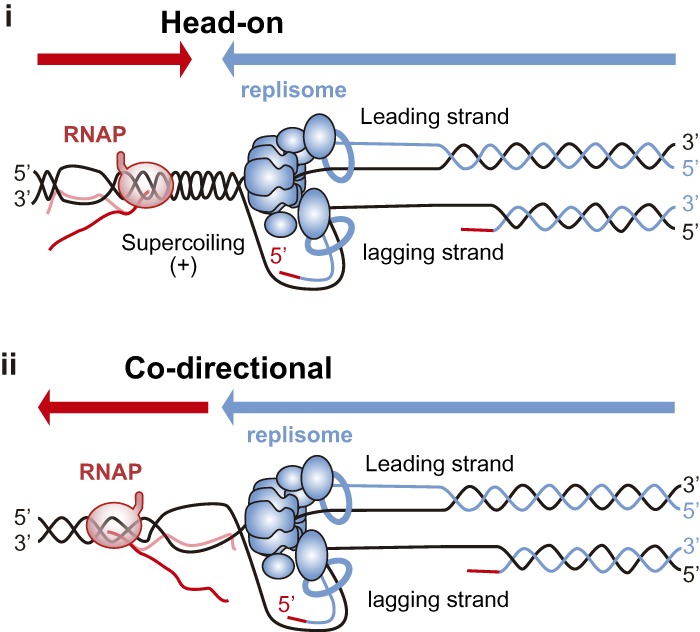
Head-on versus codirectional T–R conflicts. Transcription and replication machineries can encounter each other when travelling in head-on (panel *i*) or codirectional (panel *ii*) orientation with different consequences for the cell. Whereas the RNAP embraces both DNA strands and can constitute an obstacle by itself in both orientations, other transcription-derived obstacles such as supercoiling or DNA–RNA hybrids will have different effects depending on the orientation of the conflict. (Panel *i*) Head-on T–R conflicts might be enhanced by the generation of positive supercoiling in front of both machineries, whether they are stabilized or not by the presence of a blocked RNAP and/or DNA–RNA hybrids. (Panel *ii*) Codirectional T–R conflicts are known to be less deleterious. Although the negative supercoiling accumulated behind RNAP might facilitate the formation of DNA–RNA hybrids, these would likely be dissolved by the replication fork given the 3′–5′ polarity of the eukaryotic replicative helicase.

The enhanced replication fork pausing at head-on versus codirectional T–R conflicts together with the increased genome instability detected at head-on conflicts established that head-on T–R conflicts (Fig. 3, panel i) are more harmful for both prokaryotic and eukaryotic cells (French 1992; Liu and Alberts 1995; Mirkin and Mirkin 2005; Prado and Aguilera 2005). Conclusive evidence was provided in budding yeast by showing that deletions between direct repeats were highly induced by head-on T–R conflicts but not by codirectional ones (Prado and Aguilera 2005). In bacteria, inverting the orientation of codirectional genes to make them transcribe head-on led to impaired fork progression, loss of genome integrity and cell death (Boubakri et al. 2010; Srivatsan et al. 2010). Moreover, highly transcribed rRNA and tRNA genes contain specific polar replication fork barriers that help to prevent T–R conflicts and genetic instability (Little et al. 1993; Deshpande and Newlon 1996; Takeuchi et al. 2003).

More recent studies with human cells using an episomal assay based on the highly transcribed and DNA–RNA hybrid-prone mAIRN sequence showed increased DNA damage and checkpoint activation only in head-on T–R conflicts (Hamperl et al. 2017). However, it is important to note that R loops also form at sites of codirectional T–R conflicts (Fig. 3, panel ii), although they are not harmful by themselves, as shown in wild-type yeast cells (García-Rubio et al. 2018). In bacteria, although the consequences of head-on conflicts are worse, codirectional encounters can also generate conflicts that require auxiliary replication proteins to either bypass or resolve them (Merrikh et al. 2011). Whether codirectional T–R conflicts may also lead to R-loop-mediated DNA damage in certain eukaryotic mutant backgrounds enhancing R-loop levels has not been tested.

## T–R conflicts at specific genomic regions

There are certain genomic locations that are characterized for being intrinsically difficult to replicate, such as fragile sites, the rDNA region and telomeres. These specific regions share several features such as the presence of repetitive DNA sequences, a tendency to form non-B secondary structures, late replication timing, or a heterochromatic context. Since they all undergo active transcription, it is important to acknowledge whether T–R conflicts are behind the replication hindrance at these specific locations.

### Fragile sites

Fragile sites are genomic regions that recurrently present gaps and breaks on metaphase chromosomes when cells undergo replication stress, such as that caused by low doses of the DNA polymerase inhibitor aphidicolin. Interestingly, they frequently colocalize with the chromosome rearrangements observed in tumor cells (for review, see Durkin and Glover 2007), suggesting that fragility is exacerbated in the cellular circumstances of the tumoral process. In addition to DNA breaks and gaps, fragile sites lead to aberrant mitotic structures such as ultrafine anaphase bridges (UFBs) and micronuclei (Oestergaard and Lisby 2017). So far, three kinds of fragile sites have been described: rare fragile sites (RFSs), which were initially observed as a rare mendelian inherited trait and are associated with the expansion of dinucleotid or trinucleotide repeats with the potential to form DNA secondary structures (Magenis et al. 1970); common fragile sites (CFSs), which are regions with recurrent fragility in all individuals (Glover et al. 1984); and early replicating fragile sites (ERFSs), recently defined in highly transcribed and early replicating regions (Barlow et al. 2013).

Fragility involves fork impairment, as indicated by the fact that ATR deficiency triggers the breakage of CFSs in the absence of replicative stress (Casper et al. 2002). The fact that CFSs share a propensity to form non-B DNA structures, late replication timing, scarcity of replication origins, and long genes have contributed to the current model to consider CFSs as difficult to replicate regions, which involve frequent fork collapse and few backup origins to fire. In agreement, the number of origins inversely correlates with fragility (Letessier et al. 2011). In contrast to CFSs, ERFSs locate at regions with high-transcriptional density but are early replicating and origin-rich, which initially suggested a different mechanism for their fragility. However, recent high-resolution mapping of origin initiation and DSBs after replicative stress suggests that a common mechanism involving poly-dA:dT tracts could explain both CFSs and ERFSs (Tubbs et al. 2018).

Importantly, transcription is associated with instability in all types of fragile sites. Whereas ERFSs locate at high transcriptional density regions, many CFSs map within the coding regions of long genes and do not show fragility in the absence of transcription (Helmrich et al. 2006; Smith et al. 2006). Furthermore, artificially inducing transcription of a long, late replicating gene induces its fragility (Blin et al. 2019). Long highly transcribed regions are also hotspots for copy number variants (CNVs), which indeed tend to overlap with CFSs (Wilson et al. 2015). Transcription can promote fragility by different mechanisms. Cotranscriptional DNA–RNA hybrids have been detected in RFSs such as those at the *FXN* and *FMR1* genes from patient cells from Friedreich ataxia and Fragile X syndromes (Groh et al. 2014) and in CFSs such as *FRA3B*, *FRA16D*, and *FRA7K* (Helmrich et al. 2011). Recent reports support a role for T–R conflicts in the fragility of CFS and that R loops can contribute to such conflicts. Thus, an RNaseH-sensitive fork stalling was directly visualized in vivo by labeling DNA fibers and fluorescence in situ hybridization at the *FRA16D* CFS when FANCD2 is depleted (Madireddy et al. 2016). Consistent with the fact that FANCD2 depletion causes R-loop accumulation (García-Rubio et al. 2015; Schwab et al. 2015), FANCD2 immunoprecipitation at CFSs was also reduced by RNaseH (Okamoto et al. 2018), suggesting a role for FA pathway in removing R loops at *FRA16D*.

Finally, transcription not only promotes frequent fork stalling and collapse but seems to also be responsible for the scarcity of active replication origins at CFSs (Snyder et al. 1988; Lõoke et al. 2010). Indeed, it has been shown in vitro that RNAPII can push the loaded replicative helicase before its activation (Gros et al. 2015). If the few replication origins in these regions were not sufficient to ensure their duplication, cells would reach the next cell cycle phase with unreplicated DNA that can undergo breakage. Nonetheless, transcription can also prevent fragility by advancing the replication timing to earlier S phase, giving more time to complete replication at these regions (Blin et al. 2019).

### The rDNA region

rDNA contains multiple (150–200 in yeast and up to 350 in humans) tandem repeats of the DNA coding for rRNA, with intense transcriptional activity driven by RNAPI. These RNAPI-transcribed regions are separated by long intergenic spacers that range from 2 kb in yeast to 30 kb in mammals and that contain polar replication fork barriers, which prevent collisions with head-on RNAPI-driven transcription. The yeast rDNA loci are likely the best-studied regions for T–R conflicts and constitute the less stable regions in the yeast genome (Kobayashi 2014). In yeast, replication fork barriers are driven by the binding of the Fob1 protein and require certain replisome components, such as the Tof1/Csm3 complex that counteracts the action of the PIF1 family helicase Rrm3 (Mohanty et al. 2006). Fob1-bound DNA stalls forks leading to breaks that will be normally repaired by equal sister chromatid recombination without any consequences for the cell (Kobayashi and Ganley 2005). However, in the absence of Fob1, T–R conflicts lead to hyper-recombination between the repeats, resulting in rDNA expansions and contractions as well as in the production of extrachromosomal rDNA circles (Kobayashi and Horiuchi 1996; Kobayashi 2003; Takeuchi et al. 2003). These conflicts are likely caused by the positive supercoiling accumulated in front of RNAPI and the frequent formation of DNA–RNA hybrids (Christman et al. 1988; El Hage et al. 2010). Although still not so well studied, this mechanism seems conserved in humans, in which an RNAPI transcription terminator complex can act as a replication fork barrier at the rDNA aided by the Tof1/Csm3 orthologs TIMELESS/TIPIN (Akamatsu and Kobayashi 2015).

### Telomeres

The ends of eukaryotic chromosomes, known as telomeres, represent another source of endogenous replicative stress. Telomere maintenance is mainly carried out by telomerase in many organisms, a reverse transcriptase expressed in germ and stem cells that extends the 3′ end of chromosomes to counteract the erosion caused by replication during each round of cell division. In the absence of telomerase, homologous recombination pathways are activated for the alternative lengthening of telomeres (ALT) (Apte and Cooper 2017). Telomeres are difficult to replicate regions containing all potential obstacles to replication such as heterochromatin, torsional stress, and repetitive DNA that can promote the formation of non-B DNA structures (for review, see Gilson and Géli 2007). Furthermore, as in the case of CFSs, telomeres generally replicate late and replication origins are scarce at telomeric regions (Sfeir et al. 2009).

Despite their heterochromatic structure, telomeres produce an RNAPII transcribed long noncoding RNA named TERRA (telomeric repeat-containing RNA) (Azzalin et al. 2007; Luke et al. 2008; Schoeftner and Blasco 2008). TERRA forms R loops at telomeres, which seems to have an important role in ALT pathways (Balk et al. 2013; Pfeiffer et al. 2013; Arora et al. 2014; Yu et al. 2014), through a still not fully understood mechanism that is likely related to their capability to promote recombination. Importantly, TERRA levels are specifically increased upon telomere shortening (Arnoult et al. 2012; Cusanelli et al. 2013; Porro et al. 2014) and are cell cycle-regulated in normal cells to prevent the harmful effects of telomeric R loops on replication (Graf et al. 2017). Likely related to this, stabilization of DNA–RNA hybrids by overexpression of Yra1 protein causes telomere shortening and instability in yeast (García-Rubio et al. 2018).

## Cellular response to T–R conflicts

The mechanisms to resume DNA synthesis after fork stalling are influenced by the nature of the lesion, whether it blocks both leading and lagging strands or only one of them (for review, see Yeeles et al. 2013). When repriming is possible, it ensures the continuity of DNA synthesis. However, stalled forks can also arrest, accumulating ssDNA and leading to the activation of S-phase checkpoints. Checkpoints must maintain the stability of replication forks to promote their restart. Otherwise, forks can suffer nucleolytic degradation and can even break and/or irreversibly arrest, leading to cell death.

### Repriming or arrest

Repriming was originally shown downstream from lagging strand blocks in bacteria (McInerney and O'Donnell 2004; Nelson and Benkovic 2010) and has been confirmed recently in vitro with the yeast replisome (Taylor and Yeeles 2018). Repriming the lagging strand does not seem to be difficult, since it would involve initiating a new Okazaki fragment, leaving a ssDNA gap behind. However, repriming at the leading strand implies uncoupling between parental DNA unwinding and new DNA synthesis, leaving a ssDNA gap in the leading strand. Both bacterial and eukaryotic replisomes have an inherent ability to reprime the leading strand by themselves, although inefficiently (Heller and Marians 2006; Yeeles and Marians 2011; Taylor and Yeeles 2018). In agreement, uncoupling was reported in vertebrates (Byun et al. 2005), and ssDNA gaps have been reported in both leading and lagging strands in yeast treated with UV damage (Lopes et al. 2006). The existence of a human protein that combines polymerase and primase activities (PrimPol) and promotes DNA synthesis after UV further supports that repriming can occur in the leading strand in vivo (García-Gómez et al. 2013; Mourón et al. 2013). This ensures the progression of the fork without major delays in S phase, since ssDNA gaps left behind the fork can be repaired by postreplicative repair pathways, which involve either TLS or recombination-mediated pathways such as template switching (Branzei and Foiani 2010).

**Figure 4.**
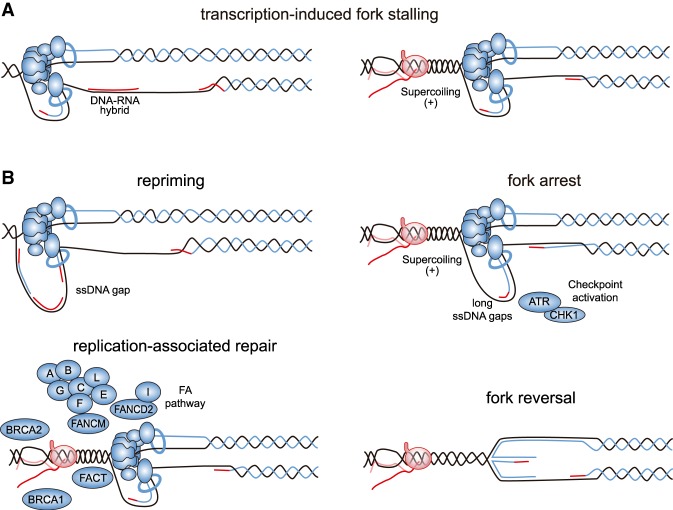
Cellular response to T–R conflicts. (*A*) The fate of a replication fork facing a transcription-induced obstacle would likely depend on the nature of the block, whether affecting only one or both replicating strands. (*B*) Possible outcomes after T–R conflicts. Repriming ahead of the fork can directly solve encounters of the lagging strand with obstacles, such as DNA–RNA hybrids. In contrast, blocks in unwinding, such as those caused by head-on T–R conflicts might induce fork reversal. Fork arrest, possibly involving some uncoupling and long ssDNA accumulation, triggers the activation of the checkpoint, which is responsible for maintaining the stability of forks, thus preventing irreversible collapse. Replication-associated repair functions, such as FACT, BRCA1, BRCA2, and FA repair pathway, are required for proper fork progression through T–R conflicts such as R loops, likely with the help of specialized helicases.

Although DNA-bound RNAP could constitute a block for both leading and lagging strands (Fig. 3) and would therefore not allow direct repriming, other transcription-derived obstacles can impose a block for only one of the replicating strands; for example, G4 or DNA–RNA hybrids (Fig. 4A). Interestingly, Prim-Pol can bypass G4 (Schiavone et al. 2014) and counteracts R-loop accumulation in human cells (Svikovic et al. 2019), suggesting that it promotes repriming after T–R conflicts driven by these non-B DNA structures (Fig. 4B). DNA–RNA hybrids could even potentially be directly used for repriming, as in mitochondria, where DNA–RNA hybrids initiate replication (Xu and Clayton 1996). Along the same line, the transcribed RNA can be used as a primer, as shown for the *E. coli* replisome in vitro (Pomerantz and O'Donnell 2008) and for the origin-independent replication recently described at the yeast rDNA locus (Stuckey et al. 2015).

When forks arrest for long enough, ssDNA accumulation triggers the activation of the S-phase checkpoints, of which the main players are the human ATR/CHK1 and yeast Mec1/Rad53 kinases (Fig. 4A; for review, see Ciccia and Elledge 2010). Checkpoint activation leads to a number of processes, some of which target the replication process itself, such as a suppression of further origin firing in order to halt S-phase progression until the replication block is released (Yekezare et al. 2013). In mammals, stringent replicative stress also triggers ATR-mediated activation of the FA pathway at the fork (Fig. 4B; Lossaint et al. 2013; Sirbu et al. 2013; Dungrawala et al. 2015). The FA-associated nuclease FAN1 is then recruited to stalled forks to restrain fork progression preventing chromosome abnormalities through a mechanism that is still not fully understood (Lachaud et al. 2016). Although T–R conflicts might elicit a specific checkpoint response, it seems that transcription-derived obstacles are sensed just as any other lesions blocking fork progression. R-loop-accumulating yeast mutants activate the S-phase checkpoint (Gómez-González et al. 2009) and head-on T–R conflicts induce ATR checkpoint activation in human cells (Hamperl et al. 2017). Furthermore, R-loop-accumulating mutants require a functional S-phase checkpoint to survive (Gómez-González et al. 2009), and an MCM-specific mutant impairing its checkpoint activation function was found to lead to R-loop-driven T–R conflicts (Vijayraghavan et al. 2016). These results suggest that the replication checkpoint protects against the harmful effect of T–R collisions. Importantly, since checkpoints constitute the main protective barrier against tumorigenesis (Bartkova et al. 2005; Gorgoulis et al. 2005), T–R conflicts might have stronger consequences in tumoral cells, where checkpoint responses are undermined.

### Fork protection and replication resumption

In the absence of a proper checkpoint response, replication forks can reverse (Sogo et al. 2002) and ultimately lead to an irreversible arrest and cell death (Lopes et al. 2001; Tercero and Diffley 2001; Tercero et al. 2003). In theory, T–R conflicts can induce fork reversal by extrusion promoted by the R loop (Fig. 4B) or by the topological barrier caused by gene-gating to the nuclear envelope. In the latter case, the checkpoint promotes the release of genes from the nuclear pore, thus specifically suppressing this deleterious effect of transcription-induced fork reversal on fork progression (Bermejo et al. 2011). Reinforcing the idea that fork reversal can be induced by T–R conflicts and R loops, a recent yeast study on large spontaneous insertions claims that some of these inserted sequences potentially arise from the cleavage of reversed forks and were enriched in R-loop-prone regions, including centromeres or telomeres (Yu et al. 2018). However, fork reversal seems a double-edge sword as it is also involved in fork stabilization in mammalian cells, thus avoiding nucleolytic degradation of the fork (for review, see Liao et al. 2018). Nucleolytic degradation is also prevented by factors such as BRCA1, BRCA2, and FANCD2 (Lomonosov et al. 2003; Schlacher et al. 2011; Ying et al. 2012; Ray Chaudhuri et al. 2016), all of which have been shown to protect from DNA–RNA hybrid accumulation, replication problems, and/or DNA breaks (Bhatia et al. 2014; García-Rubio et al. 2015; Hatchi et al. 2015; Schwab et al. 2015). These results suggest not only that fork protection and/or replication-associated repair could have a role in resolving T–R conflicts (Bhatia et al. 2017) but also that an important cause of replication fork blockage are R loops, whose dissolution by specialized pathways such as FA is critical. Indeed, the FA helicase FANCM and its yeast counterpart, Mph1, play a role in R-loop removal in vitro and in vivo (Schwab et al. 2015; Lafuente-Barquero et al. 2017). The differential role of FANCM, senataxin, and other helicases recently described to remove DNA–RNA hybrids, such as DHX9 (Chakraborty and Grosse 2011), DDX1 (Li et al. 2016), DDX19 (Hodroj et al. 2017), DDX23 (Sridhara et al. 2017), DDX21 (Song et al. 2017), or even PIF1 (Tran et al. 2017), remains to be elucidated.

Ultimately, a T–R conflict can lead to DNA breakage, which could promote replication restart via a break-induced replication-like mechanism (for reviews, see Anand et al. 2013; Yeeles et al. 2013). Replication-born DSBs are preferentially repaired by homologous recombination using the intact sister chromatid as a template (Kadyk and Hartwell 1992; Johnson and Jasin 2000; González-Barrera et al. 2003) but can also be repaired by homologous recombination with ectopic homologous sequences leading to chromosomal rearrangements (Pardo et al. 2009). Some studies suggest that transcription channels DSB repair pathway choice toward homologous recombination (Chaurasia et al. 2012; Aymard et al. 2014) and an increasing amount of reports support that transcription definitely influences DSB repair (Aguilera and Gómez-González 2017; Marnef et al. 2017). This is a phenomenon that we need to explore further, as it may have an important impact on transcription-associated genetic instability.

In conclusion, our current knowledge points to a general cellular response to replication fork blockage to warrant fork protection, avoid collapse, and allow replication resumption, with T–R conflicts being a major event triggering this response.

## Effects of T–R conflicts in genome evolution

T–R conflicts may have contributed to genome structure. In bacteria, gene disposition seems to have evolved favoring a codirectional rather than a head-on orientation of transcription with respect to replication (Merrikh 2017). Moreover, it has been proposed that the higher genetic instability of head-on oriented genes could be a motor for their faster evolution required in processes such as virulence or adaptation (Lang et al. 2017; Merrikh 2017). In eukaryotes, transcription of a number of genes seems adjusted to the temporal replication program to prevent coincidence (Meryet-Figuiere et al. 2014) and some studies mapping replication origins genome-wide have detected certain preference for T–R codirectionality in the human genome (Huvet et al. 2007; Petryk et al. 2016). Furthermore, massive transcription induced by different types of stress seems to specifically inhibit replication (Duch et al. 2013, 2018; Canal et al. 2018). However, even though preferential transcription orientation can be observed at specific locations, such as the polar replication fork barriers at rDNA and tRNA genes (Takeuchi et al. 2003), a genome-wide prevalence for T–R codirectional orientation is not as obvious as in bacteria and replication fork pauses in budding yeast have been detected at transcribed units regardless of their orientation (Azvolinsky et al. 2009). This may be due to the fact that whereas bacteria contain single well-defined replication origins and termination sites, eukaryotes contain many replication origins, with most genes having the chance to be replicated from both directions in different circumstances. Indeed, the rDNA fork barriers behave bidirectionally in humans (Little et al. 1993), likely reflecting the major flexibility of replication initiation in human cells.

Finally, the high frequency of spurious transcription observed all over genomes may suppose an important source of T–R conflicts that has not yet been properly evaluated and could have contributed to the organization of eukaryotic genomes. Indeed, deregulated long noncoding RNAs can be a potential source of unscheduled R loops and T–R conflicts, as shown in human cells depleted of the chromatin remodeler and TEF Spt6 (Nojima et al. 2018), which might explain the transcription-dependent hyper-recombination phenotype previously reported in *S. cerevisiae spt6* mutants (Malagón and Aguilera 1996). Nevertheless, recent OK-seq data in human cells have revealed that replication initiation and termination are coordinated with transcription to favor coorientation, particularly at genes occupied by high levels of RNAPII (Chen et al. 2019).

## Conclusions and perspectives

Numerous research reports over the last decades have contributed to establish transcription as a major cause of replicative hindrance. Such hindrance may not necessarily be due to the transcription machinery itself but to the consequences that transcription has on DNA structure and its surrounding chromatin. In recent years, it has become evident that transcription can lead to R-loop formation and other forms of non-B DNA structures, topological constraints or local chromatin changes, apart from potentially facilitating DNA damage. At present, an increasing number of reports are adding light to our understanding of the relevance of T–R conflicts in genome dynamics and structure. These include the identification of new factors contributing to T–R conflicts and partial deciphering of mechanisms by which cells protect stalled forks and repair replication-born DNA breaks caused by T–R collisions. However, our knowledge of the mechanisms protecting cells from the harmful effects of T–R conflicts and the mechanisms by which cells promote replication resumption or repriming after a transcription block are still scarce. We are just beginning to explore these phenomena and their relevance in cell physiology.

There are at least two aspects that need to be resolved at this point. First, do cells have specific processes, likely related to transcription, to prevent it becoming a threat for fork progression? So far, this seems to be the case, given the increasing evidence that implicates mRNP processing factors in preventing the formation of structures like R loops that enhance T–R collisions. Second, do T–R conflicts represent a particular type of harmful event for which specific resolving mechanisms have evolved or do cells just respond with the same general machinery, as in the case of transcription-independent fork-stalling threats? In the latter case, we would expect to identify known replication and repair factors, such as the FA pathway proteins with a role in solving transcription-associated replication blocks and their derived consequences. Certainly, the possibility that T–R conflicts represent a major source of replication stress and genome instability occurring in normal cells and, more significantly, in tumoral cells in which the major DNA damage response pathways are altered provides strong arguments for the need to decipher the factors and mechanisms controlling transcription-induced replication hindrance.
